# Factors Associated With Survival and Return to Function Following Synovial Infections in Horses

**DOI:** 10.3389/fvets.2019.00367

**Published:** 2019-10-22

**Authors:** Danielle E. Crosby, Raphael Labens, Kristopher J. Hughes, Sharon Nielsen, Bryan J. Hilbert

**Affiliations:** ^1^School of Animal and Veterinary Sciences, Charles Sturt University, Wagga Wagga, NSW, Australia; ^2^Sharon Nielsen Statistical Consulting and Training, Wagga Wagga, NSW, Australia

**Keywords:** horse, joint, synovial structure, outcome, prognosis

## Abstract

Synovial infections (SI) are common in horses of all ages and can be associated with high rates of morbidity and mortality. Identifying factors influencing survival and return to function may be useful for management of affected individuals and determination of prognosis. The objectives of this study were to identify factors associated with survival and return to function of horses and foals with SI presented to an equine hospital. This study is a retrospective case series. Data were collected from medical records of all horses with SI that were presented to a single equine hospital between April 1st, 2008 and May 1st, 2017. Long–term follow up was obtained by a semi-structured telephone questionnaire of clinical outcomes and analysis of online race records. Univariate models were created using generalized linear and linear mixed models to assess factors associated with outcomes. Multivariable models were created using generalized linear and linear mixed models to determine factors significantly associated with outcomes. Of 186 horses presented with SI, 161/186 (86.6%) were treated and 145/161 (90.1%) survived to discharge. The majority of joints were treated with synovial lavage (93.8%). One hundred and twenty horses were included in the return to function analysis and 79 (65%) returned to function. Increasing number of days of treatment with systemic antimicrobials was associated with increased likelihood of survival for each horse (OR 1.15, 95% CI 1.04−1.27, *P* = 0.025) and when considering each individual synovial structure (OR 1.11, 95% CI 1.04−1.17, *P* = 0.004). Horses treated with doxycycline were less likely to return to function (OR 0.39, 95% CI 0.19−0.8, *P* = 0.031). The overall rate of survival of horses treated with SI is good. The likelihood of return to function is lower than for survival. The findings of this study, combined with relevant antimicrobial stewardship practices, can be used as a part of evidence-based decision-making when veterinarians are treating horses with SI.

## Introduction

Synovial infections (SI) are common in horses of all ages. Causes of SI include direct contamination by a penetrating injury, hematogenous spread in neonates or contamination at the time of a synovial injection or surgery (1–3). Early recognition of SI and aggressive treatment are considered integral to a successful outcome (4–9). Recommended treatments for SI include high volume lavage of the affected synovial cavity to remove pathogens, toxins, inflammatory mediators, fibrin and debris combined with systemic and local or regional antimicrobial therapy (2, 10, 11). However, conventional bacterial culture and susceptibility testing of synovial fluid from horses with SI is frequently unrewarding, leading to inherent difficulty in evidence-based antimicrobial selection (1, 9, 12–16).

Synovial infections can be associated with high rates of morbidity and mortality. Reported rates of survival in horses with SI range from 42 to 98% (1, 4, 6–9, 17–25), while rates of return to function also vary widely (26–90%) (1, 4, 7–9, 20, 22, 23). Consequently, factors associated with outcome for horses with SI have been investigated, including etiology, time between synovial fluid contamination and treatment, microbiological culture results, location and number of synovial cavities affected, age, breed, treatment regimens, involvement of surrounding structures, and length of hospital stay (4, 6–9, 18, 20–23, 25–28).

A clear understanding of the factors associated with outcomes are necessary for sound treatment decisions and for formulating a more accurate prognosis for affected horses; however, a lack of agreement between existing studies makes it difficult for clinicians to estimate the effects on both survival and return to function (4, 6–9, 18, 20–23, 25–28). As such, further investigation of factors associated with outcomes of horses with SI are warranted to direct evidence-based approaches to the management and prognostic assessment of affected horses.

The objectives of this study were to (1) describe outcomes for horses and foals with SI presented to a single hospital and (2) identify the factors associated with survival to discharge from hospital and with return to function.

## Materials and Methods

All horses with SI presented to the Charles Sturt University equine hospital over a 9 year period (April 1st 2008 and May 1st 2017) were included in the study. Confirmed or suspected SI were defined as direct contamination through a breach of a joint or a tendon sheath, and/or a total nucleated cell count (TNCC) ≥30 x 10^9^ cells/L, total protein concentration ≥30 g/L and differential cell count of >80% neutrophils in a synovial fluid sample and/or a positive microbiological culture from synovial fluid (6, 7, 17, 23, 29). Microbiological culture was done by inoculating the recommended amount of synovial fluid into the BACTEC Peds Plus™/F culture bottle and this was this was immediately placed into the BACTEC 9050 automated blood culture system. All instrument positive bottles were subcultured onto agar media after bacterial growth was detected on the system. Data on signalment, history, physical findings, diagnostic tests, treatment and outcomes were retrieved from the hospital medical records. Return to function was assessed by two authors (DEC and BJH) using a semi-structured telephone questionnaire (Supplementary Item 1). In addition, for race horses, information on starts, prize money, wins, and places before and after injury were collected from online race records of Harness Racing Australia (http://www.harness.org.au/hra.cfm) for Standardbreds and Racing Australia (http://www.racingaustralia.horse) for Thoroughbreds. Survival was defined as any horse or foal that was discharged from hospital. Return to function was defined as any horse that participated in the pre-injury discipline post-injury or any horse or foal that participated in its intended discipline post-injury (both hereafter described as return to function). Charles Sturt University Human Research Ethics Committee approval (H17143) and Charles Sturt Animal Ethics Committee approval (A16065) were obtained for this study.

### Statistical Analysis

**Figure 1:**
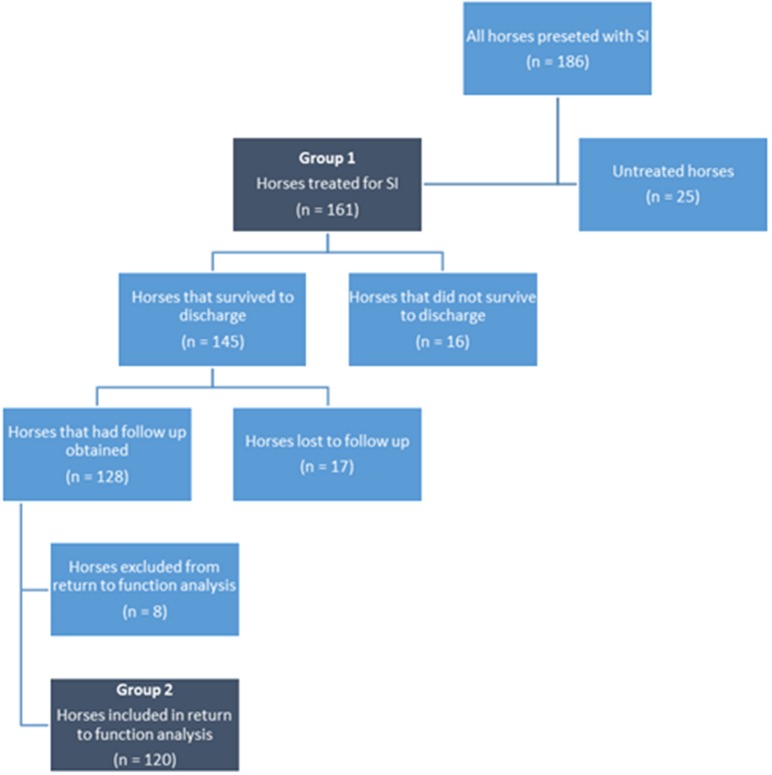
Flow diagram illustrating the process to inclusion of horses and foals.

Statistical analyses were performed for two binomial outcomes, survival and return to function (Figure 1). To determine factors associated with survival, only horses treated for SI were included. Return to function analysis included all horses that survived to discharge and were old enough to compete in their intended discipline at the time of follow up. Descriptive data were generated (using Microsoft Office Excel). For each response logistic regression was used, univariate generalized linear models (GLM) for whole horse data and generalized linear mixed models (GLMM) for synovial structure data were created prior to multivariable analyses using generalized linear and linear mixed models. Four models were used to examine explanatory variables (Supplementary Items 2, 3) specific to the individual synovial structures affected and to the horse for both outcomes of interest: Model 1 (horse level, survival, GLM) included a single explanatory variable only, Model 2 (individual synovial structure, survival, GLMM) added two random terms to Model 1—Horse ID and synovial structure within horse. Model 3 (horse level, return to function, GLM) was based on Model 1 but included multiple explanatory factors and interactions, Model 4 (individual synovial structure, return to function, GLMM) was based on Model 3 but included multiple explanatory factors and interactions. Most of the explanatory variables were qualitative but where they were quantitative a linear relationship between the explanatory variable and the response was explored. Variables from the univariate analysis with a *P***-**value of <0.1 (Supplementary Items 2, 3) were carried into the multivariable analysis. Plausible interactions between variables were investigated for both groups. For all analyses significance was set at *P* < 0.05 (statistical software R and ASRemlR was used), goodness of fit and over-dispersion were checked and were satisfactory for all models, finally, odds ratios are reported.

## Results

One hundred and eighty-six horses and foals with SI were included in the study. Of these 94 (50.5%) were referred to the hospital by a veterinarian. Horses ranged from 1 day to 25 years of age. There were 52/186 (28%) that were <6 months of age and 134/186 (72%) that ≥6 months of age. The most represented breeds were Thoroughbreds 64/186 (34.4%) and Standardbreds 41/186 (22%). Other breeds included Australian stock horses (*n* = 21), Quarter horses (*n* = 18), cross breeds (*n* = 11), Warmbloods (*n* = 7), Polo ponies (*n* = 6), Clydesdales (*n* = 4), Appaloosas (*n* = 4), pony breeds (*n* = 4), Arabians (*n* = 3), Andalusians (*n* = 2), and Miniature horses (*n* = 1). Ninety seven (52.2%) horses were female, 49/186 (26.3%) were male, 39/186 (21%) were male castrates and sex was not recorded for one horse. Function or intended function was most often racing: 47/186 (25.3%) were Thoroughbred race horses and 37/186 (19.8%) were Standardbred race horses. The functions or intended functions for all horses is provided in **Table 2**.

In total, 240 synovial structures were affected. One hundred and fifty nine (85.5%) horses had one structure affected, 18 had two structures affected, three had three structures affected, one had four structures affected, two had five structures affected, two had six structures affected, and one had ten structures affected. The forelimb was involved for 99 SI (41.3%) and the hind limb in 141 (58.7%) of SI. There were 173/240 (72.1%) joints, 50 (20.8%) tendon sheaths and 17 (7.1%) bursae affected by SI (Table 1).

**Table 1:** Frequency of distribution of infected synovial cavities.

**Synovial cavity**	**Number affected (%)**
Calcaneal bursa	8 (3.3)
Carpal sheath	3 (1.3)
Cubital joint	6 (2.5)
Digital flexor tendon sheath	42 (17.5)
Distal interphalangeal joint	17 (7.1)
Distal intertarsal joint	1 (0.4)
Extensor carpi radialis sheath	2 (0.8)
Femoropatella joint	23 (9.6)
Femorotibial joint (medial or lateral)	5 (2.1)
Middle carpal joint	16 (6.7)
Fetlock joint	36 (15.0)
Navicular bursa	9 (3.8)
Proximal interphalangeal joint	9 (3.8)
Proximal intertarsal joint	1 (0.4)
Antebrachiocarpal joint	13 (5.4)
Glenohumeral joint	2 (0.8)
Tarsal sheath	3 (1.3)
Tarsocrural joint	41 (17.1)
Tarsometatarsal joint	3 (1.3)
Total	240

The majority of cases were caused by penetrating wounds 122/186 (65.6%), followed by haematogenous spread in foals <6 months of age 42/186 (22.6%), 17 horses had idiopathic SI or haematogenous spread and five were due to an iatrogenic cause. There was a positive microbiological culture in 67/122 (54.9%) of cases and the most frequently isolated bacteria were Streptococcus species (*n* = 13), Pasteurellaciae species (*n* = 10), Staphylococcus species (*n* = 10), Escherichia species (*n* = 7), Enterococcus species (*n* = 5), and Clostridium species (*n* = 4).

Twenty-four horses (12.9%) were subjected to euthanasia after the diagnosis was made. This was due to predicted poor prognosis (*n* = 20), the cost associated with treatment (*n* = 3), and poor horse temperament for treatment (*n* = 1).

One hundred and sixty-one horses underwent treatment for SI involving 208/240 (86.7%) synovial structures. Thirteen synovial structures were treated with medical therapy only, predominantly due to client financial constraints. One hundred and ninety-five (93.8%) were treated with a form of synovial lavage: including needle lavage in 75 (38.5%) structures, endoscopic lavage in 90 (46.2%) structures, arthrotomy in six structures and in 24 (12.3%) a combination of needle lavage and endoscopic lavage or arthrotomy was used. In cases treated with synovial lavage the number of lavages ranged from 1 to 5. One hundred and forty-two (68.3%) had one lavage and 53 structures (27.2%) had more than one lavage.

All horses were treated with systemic antimicrobial therapy. The median duration of systemic antimicrobial therapy was 12 (1–77) days. Regional antimicrobial therapy was done in 127/208 of affected synovial structures (61.1%): intravenous regional perfusion of antimicrobials (*n* = 115; 90.6%), intraosseous regional perfusion of antimicrobials (*n* = 9), or a combination of intravenous and intraosseous regional perfusion (*n* = 3). Intravenous or intraosseous regional perfusion was done once in 11, twice in 28, three times in 59, four times in 15, five times in nine, and six times in five affected structures. Intrathecal administration of antimicrobials was done in 140/208 (67.3%) SI. Intrathecal administration of antimicrobials was done twice in 36 SI, three times in 24, four times in four, and five times in three.

Complications associated with treatment were observed in 15 horses, including laminitis (*n* = 2), antimicrobial-associated diarrhea (*n* = 8), colic (*n* = 2), thrombophlebitis (*n* = 1), right dorsal colitis (*n* = 1), and nosocomial wound infection (*n* = 1).

### Survival to Discharge

One hundred and forty-five (90.1%) horses survived to discharge. Of the horses <6 months of age, 36/43 (83.7%) survived to discharge and 109/118 (92.4%) of horses ≥6 months survived to discharge. Survival of horses by use is presented in Table 2.

**Table 2:** Survival and return to function of horses by use.

**Horse use or intended use**	**All horses with SI *N* (%)**	**Treated horses *N* (%)**	**Survived to discharge N (%)**	**Returned to function *N* (%)**
All	186	161/186 (86.6)	145/161 (90.1)	78/120 (65.8)
**Racehorses**	**84/186 (45.2)**	**74/84 (88.1)**	**64/74 (86.5)**	**30/60 (50)**
Thoroughbred	47/84 (25.3)	43/47 (91.5)	39/43 (90.7)	18/36 (50)
Standardbred	37/84 (19.9)	31/37 (83.8)	25/31 (80.6)	12/24 (50)
**Performance horses**	**54/186 (29)**	**47/54 (87)**	**45/47 (95.7)**	**25/32 (78.1)**
Campdrafting and western sports	16/54 (8.6)	12/16 (75)	11/12 (91.7)	7/8 (87.5)
Dressage	9/54 (4.8)	7/9 (77.8)	6/7 (85.7)	2/3 (66.7)
Endurance	2/54 (1.1)	2/2 (100)	2/2 (100)	1/1 (100)
Eventing	3/54 (1.6)	3/3 (100)	3/3 (100)	3/3 (100)
Harness	2/54 (1.1)	2/2 (100)	2/2 (100)	2/2 (100)
Polo & polo crosse	13/54 (7)	13/13 (100)	13/13 (100)	5/7 (71.4)
Rodeo	2/54 (1.1)	1/2 (50)	1/1 (100)	0/1 (0)
Show horses	3/54 (1.6)	3/3 (100)	3/3 (100)	1/3 (33.3)
Show jumping	4/54 (2.2)	4/4 (100)	4/4 (100)	4/4 (100)
**Non-performance horses**	**48/186 (25.8)**	**40/48 (83.3)**	**36/40 (90)**	**23/28 (82.1)**
General purpose	11/48 (5.9)	10/11 (90.9)	9/10 (90)	7/9 (77.8)
Broodmares	15/48 (8.1)	13/15 (86.7)	13/13 (100)	12/12 (100)
Breeding stallions	2/48 (1.1)	1/2 (50)	1/1 (100)	1/1 (100)
Paddock companions	2/48 (1.1)	1/2 (50)	1/1 (100)	1/1 (100)
Unknown	18/48 (9.7)	15/18 (83.3)	12/15 (80)	2/5 (40)

*The text in bold refers to the total numbers of horses grouped by differing athletic requirements*.

Univariate analyses revealed that increasing number of systemic treatment days (OR 1.15, 95% CI 1.04–1.27, *P* = 0.007) and the use of regional antimicrobial therapy (OR 4.42, 95% CI 1.43–13.71, *P* = 0.043) were associated with increased likelihood of survival, while an increasing number of synovial structures affected was associated with decreased likelihood of survival (OR 0.67, 95% CI 0.47–0.94, *P* = 0.029). The results of all variables examined in the univariate analysis for survival are provided in Supplementary Items 2, 3.

In the final multivariable models, an increasing number of systemic treatment days was associated with an increased likelihood of survival: model 1 (OR 1.15, 95% CI 1.04–1.27, *P* = 0.025) and Model 2 (OR 1.11, 95% CI 1.04–1.17, *P* = 0.004) (Table 3). There were no significant interactions between variables present.

**Table 3:** Multivariable analysis for survival and return to function.

**Variable**	***P*-value**	**Odds ratio**	**95% Confidence interval**
**Model 1—Horse data for survival**			
Number of days (total) on systemic antimicrobials	0.025	1.15	1.04–1.27
**Model 2—Individual synovial structure data for survival**			
Number of days (total) on systemic antimicrobials	0.004	1.11	1.04–1.17
**Model 4—Individual synovial structure data for return to function**			
Doxycycline	0.031	0.39	0.19–0.8

### Return to Function

Of the 145 individuals that survived, 17 were lost to follow up and eight were excluded from the analysis of athletic performance due to insufficient age for athletic use at the time of follow up. One hundred and twenty horses were available for long-term follow up and 78 (65%) returned to function. Ninety-two horses were used as athletes and 55/92 (58.8%) returned to function, this included 30/60 (50%) of racehorses and 25/32 (78.1%) of performance horses. The median time of follow up was 1486.5 (142–3,256) days. Of the horses <6 months of age, 16/30 (53.3%) went on to intended function while 62/90 (68.9%) of horses ≥6 months of age returned to function. Return to function of horses by use is provided in Table 2. Of racehorses that survived to discharge Standardbreds had a greater median number of starts (33 vs. 8.5), wins after injury (2 vs. 0), places after injury (6 vs. 1) and prize money after injury ($12,124 vs. $3,742.5) compared to Thoroughbreds (Table 4). Of the 42 horses that did not return to function, there were 28 race horses and 14 from other disciplines. Of the 14 horses from other disciplines 11 did not return to function due to the synovial infection, one mare was retired to stud immediately after the injury and data was lost for follow-up of two horses. Of this group, four horses are known to have been subjected to euthanasia after discharge (antimicrobial associated colitis *n* = 2, laminitis *n* = 1, lameness refractory to pain relief *n* = 1). Of the 28 race horses, there were 18 Thoroughbreds and 10 Standardbreds that did not return to function. Stud book records showed that 4/18 Thoroughbreds and 2/10 Standardbreds were recorded as deceased at the time of manuscript preparation. Additionally one Standardbred was noted to have been subjected to euthanasia after discharge, due to gastric rupture unrelated to the synovial infection.

**Table 4:** Descriptive data on return to function for racehorses that survived to discharge.

**Return to function**		**Starts before injury**		**Starts after injury**		**Wins before injury**		**Wins after injury**		**Places before injury**		**Places after injury**		**Prize money before injury (AUD)**^**c**^		**Prize money after injury (AUD)**^**c**^	
	**Mean *N* (%)**	**Median**	**Range**	**Median**	**Range**	**Median**	**Range**	**Median**	**Range**	**Median**	**Range**	**Median**	**Range**	**Median**	**Range**	**Median**	**Range**
All	30/60 (50)	0	0–50	13	1–55	0	0–9	1	0–9	0	0–16	2	0–18	$0	$0–$53,594	$7,160	$0–$59,859
TB^a^	18/36 (50)	0	0–14	8.5	1–32	0	0–3	0	0–3	0	0–3	1	0–8	$0	$0–$37,990	$3742.5	$0–$59,110
SB^b^	12/24 (50)	0	0–50	33	7–55	0	0–9	2	0–9	0	0–16	6	0–18	$0	$0–$53,594	$12,124	$645–$59,859

a Thoroughbred.

b Standardbred.

c *Australian Dollars*.

Increasing number of hospitalization days (OR 0.9, 95% CI 0.84–0.97, *P* = 0.006), treatment with doxycycline (OR 0.32, 95% CI 0.14–0.71, *P* = 0.005), an increasing number of different antimicrobial drugs administered (OR 0.63, 95% CI 0.04–0.99, *P* = 0.04), complications associated with treatment (OR 0.17, 95% CI 0.03–0.88, *P* = 0.023), increasing number of repeat treatments with intra-thecal antimicrobials (OR 0.76, 95% CI 0.62–0.93, *P* = 0.002) and an increasing number of repeated treatments with intra-thecal and regional antimicrobials (OR 0.57, 95% CI 0.41–0.8, *P* = 0.027) were negatively associated with return to function. The results of all the univariate analysis for return to function are provided in Supplementary Items 2, 3.

In the multivariable analysis, for return to function when considering each individual synovial structure (Model 4), treatment with doxycycline was negatively associated with return to function (OR 0.39, 95% CI 0.19–0.8, *P* = 0.31) (Table 3). There were no significant outcomes for the multivariable analysis of return to function when considering each horse (Model 3). Both models for return to function had no significant interactions between variables.

## Discussion

Management of septic synovial structures in horses can represent a diagnostic and therapeutic challenge. The results of this study contribute to the current body of work (1, 4, 6–9, 18, 20–23, 25–27) by introducing new clinical variables that might be helpful in making more informed treatment decisions and accurate prognostications.

In this study, 90.1% of horses treated for SI survived to discharge and of these, approximately two thirds returned to function. Specifically, the positive association between antimicrobial treatment duration with survival and doxycycline treatment being a negative prognostic indicator for return to function represent new insights.

We found positive association between longer systemic antimicrobial treatment duration and predicted survival. This may reflect an increased owner commitment to treatment; however, in a previous study, 12 days or more systemic antimicrobial treatment was associated with reduced survival and post-operative performance of horses with SI (23). Differences in reported survival rates may be a reflection of different horse populations, lesion types or other associated factors which were included in the multivariable analyses. The decreased likelihood of return to function with prolonged antimicrobial use in the univariate analyses of the current study is consistent with the findings of a previous study (23). This finding may reflect that prolonged antimicrobial use is more likely in horses with refractory infections that can be at increased risk of comorbidities or orthopedic sequelae of SI leading to reduced likelihood of return to function (9, 18, 23). As such, these associations should be considered when management of horses with SI requires prolonged administration of systemic antimicrobial drugs. The judicious use of antimicrobials must be balanced against the requirement to treat the presenting clinical condition (30, 31). According to current antimicrobial stewardship recommendations, treatment should be based on culture and *in vitro* sensitivity results, with preference made to first line antimicrobials (30, 31). Selection of antimicrobials of medical importance should be restricted to cases when infection with an organism is likely to be susceptible based on culture and sensitivity results or for the treatment of life threatening conditions (30, 31). When *in vitro* culture and sensitivity results are not available, treatment choices should be made using an evidence based approach (32).

Treatment of horses with doxycycline in our study negatively affected return to function. This is likely explained by the use of this drug in juvenile horses and/or those with infections refractory to treatment with alternative antimicrobial drugs preferentially receiving this drug. Doxycycline is readily distributed into synovial fluid at high concentrations (33). This favorable pharmacokinetic property, and the ease of oral administration of the drug for prolonged treatment durations, make doxycycline an attractive choice for treatment of SI. In addition, the likelihood of antimicrobial-associated diarrhea in foals is considered lower than in adult horses possibly due to the increased bioavailability of orally administered antimicrobials and poorly developed intestinal microflora (34, 35). These factors may influence preferences for antimicrobial treatment of foals and horses with refractory infections with doxycycline. Additionally, in this and previous studies, foals had a reduced rate of return to function (7, 21, 26) in comparison to adult horses. In the univariate analysis of the current study, increasing age was associated with increased predicted return to function. Interactions between doxycycline and both age and complications associated with treatment were investigated and were not significant.

The rate of survival of horses with SI in this study was 90.1%. This is comparable with recent reports of survival rates for horses with SI of 84–90.3% (6, 17, 18, 23), including a study from a similar geographical location (south-eastern Australia) where 84% of horses survived (6). Initial reports had lower rates of survival (65%) (36) and a poor prognosis for affected horses was described (37). With the advent of improved diagnostic techniques (13) allowing for more accurate identification of causative microorganisms and the development of endoscopic surgical techniques (18, 23) an improvement in rates of survival has been observed.

The rate of return to function for horses treated with SI in this study was 65%. Similar rates of return to athletic use (54–56.5%) have been reported in earlier studies (1, 6). Wright et al. reported a greater return to preoperative level of performance in horses with SI (81%) (23); however, their study population had a smaller proportion of foals in comparison to our study. The rate of return to function for foals (53.3%) in this study was lower than adult horses (68.9%) and other studies have similarly reported a low return to function rates for foals of 26% (7) and 48% (21). Collectively these findings suggest that adult horses have improved rates of return to function compared to foals after treatment of SI. This difference in outcome may be due to more complex clinical conditions in neonates which are more likely to have multi-systemic disease and multiple joint involvement. Both multiple SI and multi-systemic disease have previously been identified as being negatively associated with survival (7, 9, 21) and return to function (7). Although multiple SI was not retained in the final models for survival and return to function in this study, a negative association was present in the univariate analysis for survival.

Nearly half of the horses in this study population were used or intended to be used for racing (45.4%) and of the 58 race horses treated for SI available for follow up, 31 (53.5%) had at least one start in a race. This finding indicates that return to full athletic function after SI is possible. Previous rates of return to racing range between 31 and 56% (1, 7, 21), similar to the findings of the current study. After treatment of SI, Standardbreds in this study performed better than Thoroughbreds. Similar to our findings, Standardbred horses have been reported in previous studies to have increased post SI performance in comparison to Thoroughbreds with 45–62.8 and 31–36% of horses returning to racing respectively (1, 7). Although not significant in the final model, the performance data suggest an effect of breed on improved return to function. We speculate that Standardbreds in our population are an inherently more robust breed of horse that are more likely to perform despite secondary degenerative effects of SI and they also are more likely to have a greater number of lifetime starts than Thoroughbreds meaning there are more opportunities for performance outcomes.

No associations were observed between positive culture of synovial fluid and either outcome in the current study. These findings are similar to those of a previous retrospective study (1) and may be explained by the limited sensitivity of culture techniques for detection of bacteria in synovial fluid (1, 13, 27). However, a number of studies have found an association between positive bacterial culture (6, 27) or culture of specific pathogens, namely *Staphylococcus aureus* (27) and Salmonella (7) and survival or return to function in horses with SI. Positive microbiological culture may be an unreliable prognostic indicator.

In this study, increasing time between onset of SI and treatment did not have an effect on survival or return to function. Similarly, no influence of time period prior to treatment and outcome was found in a number of previous studies (6, 18–20, 28). Conversely, a delay from time of onset of SI to treatment has been identified as a negative prognostic indicator of SI in other studies (4, 5, 7–9). Case by case differences in factors such as the causative organism of SI or host immune status and other considerations including advances in treatment methods may be responsible for the conflicting findings between studies. In addition, in this study there was no association between after–hours anesthetic induction and either outcome. Previously a negative association in horses with SI between anesthetic induction outside of working hours and outcome has been reported (18). The findings of this and previous studies may suggest immediate surgical treatment is not required for positive outcomes (6, 18–20, 28).

No association was found between outcomes and bone or tendon involvement in this study. Involvement of bone and tendon with SI has been related to decreased likelihood of survival (4) and return to function (6, 9, 19). Damage to bone or tendon may lead to persistent tissue contamination or result in secondary degenerative changes increasing the likelihood of negative outcomes (4, 7, 11). The findings of this study were unexpected and may give clinicians assurance to treat horses with underlying bone and tendon involvement.

The use of, and methods for, regional antimicrobial delivery in the current study were not significantly associated with outcome in the final multivariable models; however, there was an association between regional antimicrobial therapy and survival in the univariate analysis. The local delivery of high antimicrobial concentrations is considered to be of benefit in the management of SI in horses and positively influences case outcomes (16, 38, 39). In contrast to our findings, one study found an association between regional intravenous administration of antimicrobial drugs and non-survival or reduced postoperative performance (23). The authors of that study speculated that the procedure was often used in established infections and consequently poor outcomes may reflect a bias toward those cases rather than regional therapy being an unsuccessful treatment modality (23). Despite the lack of an association in the final model between regional antimicrobial therapy and survival, this technique is likely an important part of the therapeutic regimen for treatment of SI.

In this study there was no significant effect of lavage method on outcomes. Synovial lavage is integral to the treatment of SI and endoscopy is considered the gold standard method for direct management of affected structures (2, 10, 11). This technique best allows examination of the synovial cavity (23), debridement of damaged tissue and inflammatory debris and complete lavage of the affected structure (10). The findings of this study suggest that high volume lavage may be the most important component of therapy, and this has been observed by another author (9). Repeat synovial lavage has been shown to be a negative prognostic indicator (4, 18). However, in this study more than one lavage was not associated with survival or return to function. While this finding is unexpected, it is encouraging for clinicians treating cases refractory to the initial synovial lavage.

There are limitations of the current study. The retrospective design led to some missing data that may have influenced the outcomes of data analysis. Bias due to treatment preferences of individual clinicians, horses subjected to euthanasia for reasons other than poor prognosis, horses that did not return to function for causes other than SI and possible retirement of sound horses that had greater value for breeding than as athletes were also likely. Follow up data on non-racing horses were collected directly from clients and recall bias forms another potential limitation affecting the accuracy of our data.

In conclusion, the results of this study indicate that horses with SI that are treated have a good prognosis for survival and moderate prognosis for achieving the desired use. In our study, survival was influenced by the duration of antimicrobial treatment, with a positive effect for increasing days of therapy. Return to function was negatively associated with treatment with doxycycline and horses treated with this antimicrobial drug were 2.5 times less likely to return to function. These findings provide veterinarians with information for evidence-based decision making when managing horses and foals with SI and should be combined with responsible antimicrobial stewardship. The intended outcome of effective treatment of SI must consider the judicious use of antimicrobials in order to contribute to minimizing the emergence of antimicrobial resistance.

## Data Availability Statement

The data that supports the findings of this study are available from the corresponding author upon reasonable request.

## Ethics Statement

The studies involving human participants were reviewed and approved by Charles Sturt University Human Research Ethics Committee approval (H17143). Written informed consent for participation was not required for this study in accordance with the national legislation and the institutional requirements. The animal study was reviewed and approved by Charles Sturt Animal Ethics Committee approval (A16065). Written informed consent for participation was not obtained from the owners because retrospective review of medical records with Charles Sturt Animal Ethics Committee approval (A16065).

## Author Contributions

DC contributed to study design, study execution, data analysis, and interpretation and preparation of the manuscript. RL, KH, and BH contributed to study design, study execution, interpretation and preparation of the manuscript. SN contributed to data analysis and interpretation and preparation of the manuscript.

### Conflict of Interest

SN of Sharon Nielsen Statistical Consulting and Training was employed by the School and Animal and Veterinary Sciences, Charles Sturt University. The remaining authors declare that the research was conducted in the absence of any commercial or financial relationships that could be construed as a potential conflict of interest.

## References

[1] Schneider RK Bramlage LR Moore RM Mecklenburg LM Kohn CW Gabel AA. A retrospective study of 192 horses affected with septic arthritis/tenosynovitis. Equine Vet J. (1992) 24:436–42. 10.1111/j.2042-3306.1992.tb02873.x 1459056

[2] Richardson DW Ahern BJ Synovial and osseous infections. In: Stick JA, editor. Equine Surgery. 4 Edn Saint Louis, MI: W.B. Saunders (2012). p. 1189–201. 10.1016/B978-1-4377-0867-7.00085-5

[3] Steel CM Pannirselvam RR Anderson GA. Risk of septic arthritis after intra-articular medication: a study of 16,624 injections in Thoroughbred racehorses. Aust Vet J. (2013) 91:268–73. 10.1111/avj.12073 23782019

[4] Findley JA Pinchbeck GL Milner PI Bladon BM Boswell J Mair TS. Outcome of horses with synovial structure involvement following solar foot penetrations in four UK veterinary hospitals: 95 cases. Equine Vet J. (2014) 46:352–7. 10.1111/evj.12124 23789739

[5] Herdan CL Acke E Dicken M Archer RM Forsyth SF Gee EK. Multi-drug-resistant *Enterococcus* spp. as a cause of non-responsive septic synovitis in three horses. N Z Vet J. (2012) 60:297–304. 10.1080/00480169.2011.651702 22506887

[6] Walmsley EA Anderson GA Muurlink MA Whitton RC. Retrospective investigation of prognostic indicators for adult horses with infection of a synovial structure. Aust Vet J. (2011) 89:226–31. 10.1111/j.1751-0813.2011.00720.x 21595644

[7] Steel CM Hunt AR Adams PL Robertson ID Chicken C Yovich JV. Factors associated with prognosis for survival and athletic use in foals with septic arthritis: 93 cases (1987-1994). J Am Vet Med Assoc. (1999) 215:973–7. 10511863

[8] Fraser BS Bladon BM. Tenoscopic surgery for treatment of lacerations of the digital flexor tendon sheath. Equine Vet J. (2004) 36:528–31. 10.2746/0425164044877396 15460078

[9] Wereszka MM White NA II Furr MO. Factors associated with outcome following treatment of horses with septic tenosynovitis: 51 cases (1986-2003). J Am Vet Med Assoc. (2007) 230:1195–200. 10.2460/javma.230.8.1195 17501662

[10] McIlwraith CW Nixon AJ Wright IM Endoscopic surgery in the management of contamination and infection of joints, tendon sheaths, and bursae. In: McIlwraith CW Nixon AJ Wright IM, editors. Diagnostic and Surgical Arthroscopy in the Horse. 4 Edn St. Louis, MO: Mosby (2015). p. 407–18. 10.1016/B978-0-7234-3693-5.00014-X

[11] Orsini JA Meta-analysis of clinical factors affecting synovial structure infections and prognosis. J Equine Vet Sci. (2017) 55:105–14. 10.1016/j.jevs.2017.01.018

[12] Madison JB Sommer M Spencer PA. Relations among synovial membrane histopathologic findings, synovial fluid cytologic findings, and bacterial culture results in horses with suspected infectious arthritis: 64 cases (1979-1987). J Am Vet Med Assoc. (1991) 198:1655–61. 2061187

[13] Dumoulin M Pille F van den Abeele AM Boyen F Boussauw B Oosterlinck M. Use of blood culture medium enrichment for synovial fluid culture in horses: a comparison of different culture methods. Equine Vet J. (2010) 42:541–6. 10.1111/j.2042-3306.2010.00091.x 20716195

[14] Dumoulin M Pille F Van den Abeele AM Haesebrouck F Oosterlinck M Gasthuys F. Evaluation of an automated blood culture system for the isolation of bacteria from equine synovial fluid. Vet J. (2010) 184:83–7. 10.1016/j.tvjl.2009.01.006 19230729

[15] Frees KE Lillich JD Gaughan EM DeBowes RM. Tenoscopic-assisted treatment of open digital flexor tendon sheath injuries in horses: 20 cases (1992-2001). J Am Vet Med Assoc. (2002) 220:1823–7. 10.2460/javma.2002.220.1823 12092956

[16] Lescun TB Vasey JR Ward MP Adams SB. Treatment with continuous intrasynovial antimicrobial infusion for septic synovitis in horses: 31 cases (2000-2003). J Am Vet Med Assoc. (2006) 228:1922–9. 10.2460/javma.228.12.1922 16784387

[17] Cousty M David Stack J Tricaud C David F. Effect of arthroscopic lavage and repeated intra-articular administrations of antibiotic in adult horses and foals with septic arthritis. Vet Surg. (2017) 46:1008–16. 10.1111/vsu.12696 28771839

[18] Milner PI Bardell DA Warner L Packer MJ Senior JM Singer ER. Factors associated with survival to hospital discharge following endoscopic treatment for synovial sepsis in 214 horses. Equine Vet J. (2014) 46:701–5. 10.1111/evj.12212 24417437

[19] Stewart AA Goodrich LR Byron CR Evans RB Stewart MC. Antimicrobial delivery by intrasynovial catheterisation with systemic administration for equine synovial trauma and sepsis. Aust Vet J. (2010) 88:115–23. 10.1111/j.1751-0813.2010.00553.x 20402698

[20] Smith LJ Mellor DJ Marr CM Mair TS. What is the likelihood that a horse treated for septic digital tenosynovitis will return to its previous level of athletic function? Equine Vet J. (2006) 38:337–41. 10.2746/042516406777749155 16866201

[21] Smith LJ Marr CM Payne RJ Stoneham SJ Reid SW. What is the likelihood that Thoroughbred foals treated for septic arthritis will race? Equine Vet J. (2004) 36:452–6. 10.2746/0425164044868396 15253089

[22] Post EM Singer ER Clegg PD Smith RK Cripps PJ. Retrospective study of 24 cases of septic calcaneal bursitis in the horse. Equine Vet J. (2003) 35:662–8. 10.2746/042516403775696285 14649357

[23] Wright IM Smith MR Humphrey DJ Eaton-Evans TC Hillyer MH. Endoscopic surgery in the treatment of contaminated and infected synovial cavities. Equine Vet J. (2003) 35:613–9. 10.2746/042516403775467225 14515964

[24] Meijer MC van Weeren PR Rijkenhuizen AB. Clinical experiences of treating septic arthritis in the equine by repeated joint lavage: a series of 39 cases. J Vet Med A Physiol Pathol Clin Med. (2000) 47:351–65. 10.1046/j.1439-0442.2000.00290.x 11008444

[25] Vos NJ Ducharme NG. Analysis of factors influencing prognosis in foals with septic arthritis. Ir Vet J. (2008) 61:102–6. 10.1186/2046-0481-61-2-102 21851707 PMC3113883

[26] Wright L Ekstrøm CT Kristoffersen M Lindegaard C Haematogenous septic arthritis in foals: short- and long-term outcome and analysis of factors affecting prognosis. Equine Vet Educ. (2017) 29:328–36. 10.1111/eve.12616

[27] Taylor AH Mair TS Smith LJ Perkins JD. Bacterial culture of septic synovial structures of horses: does a positive bacterial culture influence prognosis? Equine Vet J. (2010) 42:213–8. 10.2746/042516409X480403 20486977

[28] Kidd JA Barr AR Tarlton JF. Use of matrix metalloproteinases 2 and 9 and white blood cell counts in monitoring the treatment and predicting the survival of horses with septic arthritis. Vet Rec. (2007) 161:329–34. 10.1136/vr.161.10.329 17827471

[29] Steel CM. Equine synovial fluid analysis. Vet Clin N Am Equine Pract. (2008) 24:437–54. 10.1016/j.cveq.2008.05.004 18652964

[30] Weese JS Giguère S Guardabassi L Morley PS Papich M Ricciuto DR. ACVIM consensus statement on therapeutic antimicrobial use in animals and antimicrobial resistance. J Vet Intern Med. (2015) 29:487–98. 10.1111/jvim.12562 25783842 PMC4895515

[31] Raidal S. Antimicrobial stewardship in equine practice. Austr Vet J. (2019) 97:238–42. 10.1111/avj.12833 31236925

[32] Reed SM Bayly WM Sellon DC Equine Internal Medicine. St. Louis, MO: Elsevier Health Sciences (2017).

[33] Schnabel LV Papich MG Watts AE Fortier LA. Orally administered doxycycline accumulates in synovial fluid compared to plasma. Equine Vet J. (2010) 42:208–12. 10.2746/042516409X478514 20486976

[34] White G Prior SD. Comparative effects of oral administration of trimethoprim/sulphadiazine or oxytetracycline on the faecal flora of horses. Vet Rec. (1982) 111:316–8. 10.1136/vr.111.14.316 6293150

[35] Moore Keir AA Stampfli HR Crawford J Outbreak of acute colitis on a horse farm associated with tetracycline- contaminated sweet feed. Can Vet J. (1999) 40:718–20. 10572668 PMC1539814

[36] Gibson KT McIlwraith CW Turner AS Stashak TS Aanes WA Trotter GW. Open joint injuries in horses: 58 cases (1980-1986). J Am Vet Med Assoc. (1989) 194:398–404. 2917911

[37] McIlwraith CW. Treatment of infectious arthritis. Vet Clin N Am Large Anim Pract. (1983) 5:363–79. 10.1016/S0196-9846(17)30083-6 6356569

[38] Whitehair KJ Bowersock TL Blevins WE Fessler JF White MR Sickle DC Regional limb perfusion for antibiotic treatment of experimentally induced septic arthritis. Vet Surg. (1992) 21:367–73. 10.1111/j.1532-950X.1992.tb01713.x 1413470

[39] Rubio-Martinez LM Elmas CR Black B Monteith G. Clinical use of antimicrobial regional limb perfusion in horses: 174 cases (1999-2009). J Am Vet Med Assoc. (2012) 241:1650–8. 10.2460/javma.241.12.1650 23216042

