# Supplementary material for: Factors Associated With Survival and Return to Function Following Synovial Infections in Horses
Source: Front Vet Sci. 2019 Oct 22;6:367. doi: 10.3389/fvets.2019.00367 (PMC6817570; doi:10.3389/fvets.2019.00367)
Supplement: Supplementary file 1 [file Table_1.DOCX]

**Supplementary Item One - Telephone Questionnaire**

Hello......(Client’s name)…. My name is….(Researcher’s name)…from the Veterinary Clinical Centre, CSU. I am contacting you to ask you some questions about your horse (horse’s name) that was treated at the Veterinary Clinical Centre.

Consent: Do you consent to answer questions about (horse name) recovery from treatment of synovial infection at the Veterinary Clinical Centre? This information will be used to help evaluate and improve treatment protocols and outcomes for animals with septic joints. Information collected will have the identity of both you and your horse removed before analysis and presentation and publication of the study results. At any time during the survey period (September 2017 to March 2018) if you would like to amend your responses, or withdraw participation in the project, please do not hesitate to contact the principal researcher, using the contact details provided on the information letter.

1. Did the horse return to its previous or intended use?

YES NO

(Yes: go to Qu 2, No: go to Qu 3).

2. Did the horse return to a lower, equal or greater level of performance?

LOWER EQUAL GREATER

(Go to question 6).

3. Was the injury to the synovial structure the reason the horse did not reach its intended use?

YES NO

(Yes: go to question 5, No: go to question 4).

4. Was there any other reason the horse may not have been able to achieve its intended use?

YES NO

Notes:

(Go to question 5).

5. Was the horse used for another purpose?

YES NO

Notes:

(Go to question 6).

6. Does/did the horse require ongoing management after treatment of the infection?

YES NO

(Yes: go to Qu 7, No: go to Qu 8).

7. What treatments has the horse received for ongoing management after the infection e.g. Hyaluronic acid/ corticosteroids/ NSAIDs/other).

Notes:

(Go to Qu 8).

8. Would you like a summary of the findings of this study, when available? (End Survey).

YES NO

End - Thank-you for your time and for participating in this survey.
