# Supplementary material for: Factors Associated With Survival and Return to Function Following Synovial Infections in Horses
Source: Front Vet Sci. 2019 Oct 22;6:367. doi: 10.3389/fvets.2019.00367 (PMC6817570; doi:10.3389/fvets.2019.00367)
Supplement: Supplementary file 2 [file Table_2.DOCX]

**Supplementary item 2. Univariate analysis of categorical variables investigated for association with survival to discharge and return to function**

| **Variable** | **Survival** | | | | **Return to Function** | | | |
| --- | --- | --- | --- | --- | --- | --- | --- | --- |
|  | **Odds Ratio** | **95% Confidence Interval** | **P value** | **Included in multivariable analysis** | **Odds Ratio** | **95% Confidence Interval** | **P value** | **Included in multivariable analysis** |
|  | **Signalment & history** | | | | | | | |
| Breed. |  |  | 1 |  |  |  | 0.455 |  |
| Sex. |  |  | 0.59 |  |  |  | 0.707 |  |
| Use or intended use. |  |  | 1 |  |  |  | 0.117 |  |
| If the patient was referred. |  |  | 0.862 |  |  |  | 0.444 |  |
| Day of the week of admission. |  |  | 0.884 |  |  |  | 0.755 |  |
| Year of admission. |  |  | 0.774 |  |  |  | 0.492 |  |
|  | **Findings from clinical examination and diagnostic tests** | | | | | | | |
| Cause of SI. |  |  | 0.067 | Model 1; Model 2 |  |  | 0.17 |  |
| Type of synovial structure affected. |  |  | 0.153 |  |  |  | 0.551 |  |
| What structure was affected? |  |  | 0.944 |  |  |  | 0.927 |  |
| What limb was affected? |  |  | 0.656 |  |  |  | 0.067 |  |
| Was there significant findings related to the SI on radiographic examination? |  |  | 0.985 |  |  |  | 0.175 |  |
| Were there significant findings related to the SI on sonographic examination? |  |  | 0.853 |  |  |  | 0.846 |  |
| Gross appearance of synovial fluid on in house testing. |  |  | 0.716 |  |  |  | 0.999 |  |
| What was the gross appearance of the synovial fluid on admission on external lab testing? |  |  | 0.835 |  |  |  | 0.334 |  |
| Was cytological examination reflective of synovial sepsis? |  |  | 0.371 |  |  |  | 0.062 |  |
| Did the horse have anti-microbial therapy prior to synovial fluid culture? |  |  | 0.868 |  |  |  | 0.725 |  |
| Were microbiological organisms cultured from synovial fluid collected at admission? |  |  | 0.805 |  |  |  | 0.936 |  |
| What organism was cultured from synovial fluid? |  |  | 1 |  |  |  | 0.999 |  |
| Were cultured organisms multi-drug resistant? (Resistant to three or more antimicrobials on sensitivity testing). |  |  | 0.775 |  |  |  | 0.591 |  |
| **Treatment** | | | | | | | | |
| If the patient was hospitalised. |  |  | 0.856 |  |  |  | 0.874 |  |
| Treatment with systemic antimicrobials (at all). |  |  | 0.925 |  |  |  |  |  |
| Treatment with systemic antimicrobials prior to admission to the hospital. |  |  | 0.638 |  |  |  | 0.876 |  |
| Type of systemic anti-microbial used: | | | | | | | | |
| Procaine penicillin |  |  | 0.152 |  |  |  | 0.832 |  |
| Gentamicin |  |  | 0.152 |  |  |  | 0.728 |  |
| Doxycycline |  |  | 0.219 |  | 0.32 | 0.14-0.71 | 0.005 | Model 3; Model 4 |
| Trimethoprim sulphonamide |  |  | 0.063 | Model 1; Model 2 |  |  | 0.101 |  |
| Ceftiofur |  |  | 0.888 |  |  |  | 0.282 |  |
| Amikacin |  |  | 0.150 |  |  |  | 0.157 |  |
| Marbofloxacin |  |  | 0.950 |  |  |  | 0.157 |  |
| Cefazolin |  |  | 0.151 |  |  |  | 0.282 |  |
| Enrofloxacin |  |  | 0.641 |  |  |  | 0.53 |  |
| Oxytetracycline |  |  | 0.563 |  |  |  | 0.957 |  |
| Metronidazole |  |  | 0.089 |  |  |  | 0.495 |  |
| Chloramphenicol |  |  | 0.504 |  |  |  | 0.582 |  |
| Rifampicin |  |  | 0.950 |  |  |  | NA |  |
| Were there complications associated with treatment? |  |  | 0.199 |  | 0.17 | 0.03-0.88 | 0.023 | Model 3; Model 4 |
| Who was the surgeon? |  |  | 0.846 |  | 127634439.64 | 0, Inf | 0.017 | Model 3; Model 4 |
| Was the surgery done after hours? |  |  | 0.984 |  |  |  | 0.228 |  |
| **Structure specific treatment** | | | | | | | | |
| Was surgery done on the affected synovial structure? |  |  | 0.107 |  |  |  | 0.301 |  |
| Was a needle flush done on the affected structure? |  |  | 0.115 |  |  |  | 0.385 |  |
| Was an arthroscopy done on the affected structure? |  |  | 0.097 | Model 2 |  |  | 0.593 |  |
| Was an arthrotomy done on the affected structure? |  |  | 0.842 |  |  |  | 0.978 |  |
| Was regional antimicrobial therapy done on the affected synovial structure? | 4.42 | 1.43-13.71 | 0.043 | Model 2 |  |  | 0.857 |  |
| Was an intravenous regional perfusion done on the affected synovial structure? |  |  | 0.453 |  |  |  | 0.733 |  |
| Was an intraosseous regional perfusion done on the affected synovial structure? |  |  | 0.569 |  |  |  | 0.733 |  |
| Was an intrathecal antimicrobial administration done on the affected synovial structure? |  |  | 0.455 |  |  |  | 0.059 |  |

(SI, Synovial infections; GLM, generalised linear models; GLMM, generalised linear mixed models; Model 1 (horse level, survival, GLM); Model 2, (individual synovial structure, survival, GLMM); Model 3 (horse level, return to function GLM); Model 4 (individual synovial structure, return to function, GLMM)).
