# Supplementary material for: Factors Associated With Survival and Return to Function Following Synovial Infections in Horses
Source: Front Vet Sci. 2019 Oct 22;6:367. doi: 10.3389/fvets.2019.00367 (PMC6817570; doi:10.3389/fvets.2019.00367)
Supplement: Supplementary file 3 [file Table_3.DOCX]

**Supplementary item 3. Univariate analysis for continuous variables investigated for association with survival to discharge and return to function**

| **Variable** | **Survival** | | | | **Return to Function** | | | |
| --- | --- | --- | --- | --- | --- | --- | --- | --- |
|  | **Odds Ratio** | **95% Confidence Interval** | **P value** | **Included in multivariable analysis** | **Odds Ratio** | **95% Confidence Interval** | **P value** | **Included in multivariable analysis** |
| Age. |  |  | 0.912 |  | 1 | 1-1 | 0.042 | Model 3; Model 4 |
| Days between onset of clinical signs and admission to the hospital. |  |  | 0.192 |  |  |  | 0.347 |  |
| Number of affected synovial structures. | 0.67 | 0.47-0.94 | 0.029 | Model 1; Model 2 |  |  | 0.722 |  |
| Synovial fluid total protein (g/L) on in house testing. |  |  | 0.472 |  |  |  | 0.359 |  |
| Synovial fluid lactate (mmol/L) on in house testing. |  |  | 0.164 |  |  |  | 0.644 |  |
| Difference between systemic and synovial fluid lactate (mmol/L) on in house testing. |  |  | NA |  |  |  | NA |  |
| What was the synovial fluid glucose (mmol/L) on admission on in house testing? |  |  | NA |  |  |  | NA |  |
| What was the difference between the systemic and synovial fluid glucose on admission (mmol/L) on in house testing? |  |  | NA |  |  |  | NA |  |
| What was the synovial fluid total protein (g/L) on admission on external testing? |  |  | 0.206 |  |  |  | 0.389 |  |
| What was the synovial fluid total nucleated cell count on external testing? |  |  | 0.373 |  |  |  | 0.389 |  |
| What was the synovial fluid total red cell count on testing? |  |  | 0.507 |  |  |  | 0.519 |  |
| Number of days the patient was in hospital. |  |  | 0.263 |  | 0.9 | 0.84-0.97 | 0.006 | Model 3; Model 4 |
| Cost of treatment. |  |  | 0.636 |  | 1 | 1-1 | 0.022 | Model 3; Model 4 |
| Number of days (total) on systemic antimicrobial therapy. | 1.15 | 1.04-1.27 | 0.007 | Model 1; Model 2 | 0.97 | 0.95-1 | 0.045 | Model 3; Model 4 |
| Total number of different antimicrobials used. |  |  | 0.193 |  | 0.63 | 0.4-0.99 | 0.04 | Model 3; Model 4 |
| How many times was surgery done on the affected structure? |  |  | 0.533 |  |  |  | 0.072 |  |
| How many times was regional antimicrobial therapy done in total? |  |  | 0.609 |  |  |  | 0.888 |  |
| How many times was intra-thecal antimicrobial administration done. |  |  | 0.146 |  | 0.76 | 0.62-0.93 | 0.027 | Model 3 |
| How many times was regional and intra-thecal antimicrobial administration done in total? |  |  | 0.138 |  | 0.57 | 0.41-0.8 | 0.002 | Model 3 |

(SI, Synovial infections; GLM, generalised linear models; GLMM, generalised linear mixed models; Model 1 (horse level, survival, GLM); Model 2, (individual synovial structure, survival, GLMM); Model 3 (horse level, return to function GLM); Model 4 (individual synovial structure, return to function, GLMM)).
